# Long-term impact of farm management and crops on soil microorganisms assessed by combined DGGE and PLFA analyses

**DOI:** 10.3389/fmicb.2014.00644

**Published:** 2014-12-10

**Authors:** Fabio Stagnari, Giorgia Perpetuini, Rosanna Tofalo, Gabriele Campanelli, Fabrizio Leteo, Umberto Della Vella, Maria Schirone, Giovanna Suzzi, Michele Pisante

**Affiliations:** ^1^Faculty of BioScience and Technology for Food, Agriculture and Environment, University of TeramoMosciano Sant'Angelo, Italy; ^2^Consiglio per la Ricerca e la Sperimentazione in Agricoltura - Unità di Ricerca per l'Orticoltura (CRA - ORA)Monsampolo del Tronto, Italy

**Keywords:** organic and conventional food, microbial groups, fungal community, soil fertility indicators, long-term soil management

## Abstract

In the present study, long-term organic and conventional managements were compared at the experimental field of Monsampolo del Tronto (Marche region, Italy) with the aim of investigating soil chemical fertility and microbial community structure. A polyphasic approach, combining soil fertility indicators with microbiological analyses (plate counts, PCR-denaturing gradient gel electrophoresis [DGGE] and phospholipid fatty acid analysis [PLFA]) was applied. Organic matter, N as well as some important macro and micronutrients (K, P, Mg, Mn, Cu, and Zn) for crop growth, were more available under organic management. Bacterial counts were higher in organic management. A significant influence of management system and management x crop interaction was observed for total mesophilic bacteria, nitrogen fixing bacteria and actinobacteria. Interestingly, cultivable fungi were not detected in all analyzed samples. PLFA biomass was higher in the organic and Gram positive bacteria dominated the microbial community in both systems. Even if fungal biomass was higher in organic management, fungal PCR-DGGE fingerprinting revealed that the two systems were very similar in terms of fungal species suggesting that 10 years were not enough to establish a new dynamic equilibrium among ecosystem components. A better knowledge of soil biota and in particular of fungal community structure will be useful for the development of sustainable management strategies.

## Introduction

The market of organic foods in Europe is growing and it has evolved from a niche to the mainstream; in this contest Italy represents the fourth largest market for organics (Hughner et al., 2007). Organic agriculture was firstly defined by the European Union regulation (CEE/2092/91 and CEE1804/99) (Lairon, 2009) and it is considered to be more environmentally sound than intensive agriculture, which is dependent on the routine use of herbicides, pesticides, and inorganic nutrient for crops and animals production (Bengtsson et al., 2005). Generally, organic farmers do not use conventional inputs: pests are naturally controlled and crops are rotated and diversified (Reganold et al., 2010). In such systems, plant growth and ecosystem productivity are based on the natural availability of plant nutrients, the use of green manure and organic soil amendments (Berry et al., 2002). Several advantages, related to soil quality including higher water holding capacity, cation exchange capacity (CEC), lower bulk density, and build-up of nutrient stocks can be obtained, with a favorable impact on vegetable production (Bulluck et al., 2002). Other benefits include pH stabilization and faster water infiltration rate (Stamatiadis et al., 1999). One fundamental aspect for soil quality, which has not been appropriately considered till some years ago, is represented by “biodiversity,” normally indicated as the variability of the living forms, soil fauna, flora, vertebrates, birds, and mammals within an habitat or a management system of a territory involved in agricultural activity. A larger microbial biodiversity significantly affect all the small-scale processes that underlie many environmentally important functions such as mineralization of nitrogen, phosphorus and sulfur, as well as soil aggregates formation and pesticides degradation (Bonilla et al., 2012; Zhang et al., 2012; Lorenzo et al., 2013). However, the precise definition of soil microbial community structure, diversity and functions remains still a black box. Most soil microbes withstand laboratory cultivation so, very few have been isolated, cultured and identified, and directly related to their function in agroecosystems (Fierer and Jackson, 2006). A recent project called TerraGenoma (Vogel et al., 2009) highlighted the huge variability in soil microbial community as affected by space, time and management (organic vs. conventional), confirming the complexity of this ecosystem. In recent years, the approaches for studying soil microbiota have moved from biochemical and microbiological determinations (enzyme activities, microbial biomass and respiration coefficients) to the investigation of bacterial diversity and microbial communities structure (Hill et al., 2000). No single method can give a reliable description of the total community, consequently a combined multidisciplinary approach would offer the opportunity to correlate information, and to overcome and minimize drawbacks, arising from culture-dependent and independent methods. Moreover, to provide a more complete picture of microbial diversity and a deeper understanding of the interactions in soil microbial “sociology” statistical analyses are needed to integrate the results obtained with these methods (Reganold and Dobermann, 2012).

In light of the above, it is very important to understand the impact of the agricultural systems on microbial biodiversity and consequently on soil fertility, through the use and the definition of appropriate biological, chemical, and physical indicators (Mader et al., 2002; van Diepeningen et al., 2006; Reganold et al., 2010). To develop a sustainable agricultural model, based not only on crop productivity but also on ecological principles, long-term field experiments are necessary. Some studies have compared conventional to organic farming systems, but the obtained results are often controversial because of the interactive effects of several farming practices, soil quality, crop varieties, time of harvesting etc. (Reganold et al., 2010). Only very few long-term studies have been conducted under Mediterranean conditions. Recently, Campanelli and Canali (2012) investigated the effect of a long-term organically managed farming system for vegetable production in central Italy, on yield and quality of six species, within the project Monsampolo Organic Vegetable (MOVE).

The aim of the present study was to characterize the physico-chemical and microbiological traits of the soils from MOVE project, managed according to two different agricultural systems (organic vs. conventional) and evaluating the effect of the crop (*Brassica oleracea* L. and *Foeniculum vulgare* M.) in a 10-year field experiment. A multidisciplinary approach was applied, combining soil fertility indicators with phospholipid fatty acids analysis (PLFA) and PCR DGGE analysis (denaturing gradient gel electrophoresis).

## Materials and methods

### Field experiments and crop management

The MOVE long-term experiment started in 2001 at Monsampolo del Tronto (AP), (latitude 42° 53′ N, longitude 13°48′ E) a typical coastal area of Central Italy.

A 4-year crop rotation based on six main crops was established following conventional and organic systems. The rotation was: tomato (*Lycopersicon esculentum* Mill.), melon (*Cucumis melo* L.), fennel (*Foeniculum vulgare* M. var. azoricum), lettuce (*Lactuca sativa* L.), cauliflower (*Brassica oleracea* L. var. botrytis), and bean (*Phaseolus vulgaris* L.). Three different green manures were included in the rotation of the organic system: hairy vetch (*Vicia villosa* R.), grown before tomato transplanting, barley (*Hordeum vulgare* L.), grown before melon, and radish (*Raphanus sativus* L.), grown before lettuce.

The conventional and organic managements, located in neighboring fields of about 2 200 m^2^ each, were separated by field margins and traffic infrastructure in order to avoid any cross-contamination effect between the systems. A 5-year period of conversion (2001–2006) was observed to ensure reliable long-term production goals and to overcome the effects of short-term soil changes (Mazzoncini et al., 2010).

The soil of the conventional system was plowed to a depth of 0.4 m and the sowing or the transplanting beds were prepared by harrowing (0.2–0.3 m deep, depending on the crop). The soil of the organic system was tilled by rotary spader and successively harrowed (0.1–0.2 m deep, depending on the crop). In the conventional system, weeds were controlled by the application of pre- or post-emergence/transplantation herbicides. In the organic system, an integrated weed management approach consisting of the use of green manures and of direct control methods (false-seed-bed, shallow harrowing and manual hoeing) was employed.

Conventional areas were fertilized with synthetic and mineral NPK fertilizers, while organic crops received off-farm organic fertilizers selected in accordance to the annex I of the European regulation (European Union, 2008). In Table 1 the amount of the nutritive elements of off-farm origin applied to the two systems during the entire rotation is shown. The plots were irrigated similarly both in conventional and organic systems, by sprinkler method (bean, cauliflower, fennel, and lettuce) or drip irrigation (tomato and melon).

**Table 1:** **Nutritive elements of off-farm origin applied to the organic and conventional system of the MOVE long term trial**.

	**Organic**			**Conventional**		
**Crop**	**N**	**P**	**K**	**N**	**P**	**K**
Tomato	176	35	24	208	60	210
Melon	102	28	14	112	48	169
Fennel	177	29	45	208	39	100
Lettuce	132	15	22	130	43	136
Cauliflower	184	50	43	203	52	116
Bean	39	7	13	44	35	80
Rotation	810	164	161	905	276	811

Values are reported in kg/ha and are the mean of 3 years (2007–2010).

### Soil sampling

Soil samples were collected over the growing season 2010 on conventional and organic plots hosting cauliflower and fennels. Five replicate samples, each comprising of three replications, were taken randomly from each different block between the crop rows at one depth (0–10 cm) and immediately stored at 4°C. Stones, large pieces of plant material and soil animals were removed before use. The samples were named as follows: ORG/FEN (organic management/*Foeniculum vulgare*), ORG/CAU (organic management/*Brassica oleracea*), CON/FEN (conventional management/*Foeniculum vulgare*), CON/CAU (conventional management/*Brassica oleracea*).

### Soil chemical and physical properties

The dry weight was determined by placing 10 g of sample in an oven at 105°C. The mass was held constant when the difference in values of the weights were less than 10 mg within an hour. Soil pH was measured in a 1:1 w/v water. Physical and chemical characteristics were determined following the Official Methods of Analysis (AOAC, 2005) and were expressed as mean of three replications, each one was repeated twice.

### Microbial counts

Soil microorganisms were extracted by shaking 10 g of soil in 90 mL of peptone water (pH 7.2, Oxoid, Milan, Italy). After shaking conventional dilution spread-plating was performed to assess the total and specific cultivable bacterial and fungal communities. For this purpose the following media were prepared: Potato Dextrose Agar (PDA) for fungal count, Tryptone Soy Agar (TSA) for total bacterial count. Actinobacteria and nitrogen-fixing bacteria were grown on two selective media: starch casein agar (Kuster and Williams, 1964) and Brown's glucose agar (Thompson, 1989), respectively. Plates were incubated at 28°C for fungi and bacteria. Nitrogen-fixing bacteria, total mesophilic microorganisms and fungi were detected after 3-6-10 days, while actinobacteria after 7 days. Data from triplicate counts were expressed according to the following equation: ∑ C/∑ nz where C is the number of colonies counted on the various plates considered, regardless of dilution, z is the dilution factor (10^−x^) and n is the number of plates of each dilution (Cavalli-Sforza, 1966).

### Phospholipid fatty acid analysis (PLFA)

Lipids were extracted from soil, fractionated and quantified according to Bardgett et al. (1996). Separated fatty acid methyl esters were identified by chromatographic retention time and mass spectral comparison using bacterial acid methyl esters mix (FAME; Matreya 1114), methyl 10(Z)-heptadecenoate (Matreya 1203). The internal standard methyl non-adecanoate (Matreya 1029) was used to quantify data. Fatty acid nomenclature was used as described by Frostegard et al. (1993). The fatty acids i15:0, a15:0, 15:0, i16:0, 17:0, a17:0, i17:0, cy17:0, 16:1ϖ9c and cy19:0 were chosen to represent bacterial PLFAs (Dungait et al., 2011) while and 18:2ϖ6, 9c and 18:1ϖ9c were used as indicators of fungal community (Baath, 2003).

### Eumycetic community fingerprinting by PCR-DGGE

DNA was extracted from soil samples using PowerSoil DNA Isolation Kit (MoBio Laboratories, Inc. Carlsbad, CA, USA) according to manufacturer's instructions. Quantification of total DNA was achieved using a VersaFluor fluorimeter and a Fluorescent DNA Quantitation Kit (Bio-Rad, Milan, Italy). PCR was performed using primers FR1-GC (5′-(*GC*) AICCATTCAATCGGTAIT-3′) and FF 390 (5′-CGATAACGAACGAGACCT-3′) according to Mello et al. (2010).

Gels were stained with ethidium bromide solution (5 μg/mL; 20 min), washed with deionized water, and viewed by UV transillumination. Conversion, normalization, and further analysis of the DGGE patterns were carried out with *Fingerprinting* II Informatix™ software program (Bio-Rad). Similarities among profiles were calculated using the Unweighted Pair-Group Method with Average (UPGMA) algorithm.

### Sequencing of DGGE bands

DGGE bands of interest were excised from the gel immediately after the staining in order to identify the dominant fungal populations. DNA from selected bands was put in distilled water and re-amplified with the primers FF 390 and FR1 (without *GC*-clamp) as described above in a final PCR volume of 20 μL. The amplified fragment was then purified using GFX™ PCR DNA and Gel Band Purification Kit (Amersham Biosciences AB, Uppsala, Sweden), according to the manufacturer's instructions and after drying was delivered to BMR Genomics (Padua University, Padua, Italy) for sequencing. The obtained sequences were compared with those available at the National Center for Biotechnology Information (NCBI) using BLASTN (Altschul et al., 1990).

### Statistical analysis

Collected data regarding microbiological count, fatty acids and soil chemical and physical characteristics were subjected to two-way analysis of variance (ANOVA) using the R software (*R Foundation for Statistical Computing*, Vienna, Austria). If the ANOVA detected significant differences, means separation was conducted through the Fisher's Least Significant Difference (LSD) test. The principal component analysis (PCA) was applied to summarize correlation among treatments (management systems/crops) and variables (soil chemical and microbial characteristics) using the statistical software STATISTICA for Windows (STAT. version 8.0, StatSoft Inc. Tulsa, OK, USA).

## Results and discussion

### Soil chemical and physical properties

The physical and chemical characteristics of the soils under investigation are reported in Table 2. Generally, organic management showed significant larger values of several characteristics; organic matter content was 35% higher (17.3 vs. 12.7 g/kg in organic and conventional, respectively) as well CEC (15.3 vs. 14.9 meq 100/g in organic and conventional, respectively). Depending on soil type and climate, the capacity of a soil to store organic matter levels may be increased also in combination with the suitable soil management (Parton et al., 1996).

**Table 2:** **Chemical characteristics of soils under organic and conventional management**.

**System**	**Crop**	***pH***	***Organic matter*^†^**	***N tot*^††^**	***N nitric*^††^**	***Assimilable*^††^**					***Exchangeable*^††^**		***C/N ratio***	***Mg/K ratio***	***CEC*^†††^**
						***P***	***Fe***	***Mn***	***Cu***	***Zn***	***K***	***Mg***			
Conventional	Fennel	8.2	12.9^c^	0.8^c^	5.7^d^	25^d^	12^b^	3.6^c^	7.7^d^	1.2^c^	347^d^	191^d^	9.4^a^	1.8^b^	14.8^b^
	Cauliflower	8.3	12.6^d^	0.8^c^	9.7^c^	32^b^	10^c^	3.4^c^	11.1^b^	1.2^c^	357^c^	242^c^	9.2^b^	2.2^a^	14.9^b^
	*Overall mean*	8.3	12.8^B^	0.8^B^	7.7^B^	28^B^	11	3.5^B^	9.4^B^	1.2^B^	352^B^	217^B^	9.3^A^	2.0	14.9^B^
Organic	Fennel	8.2	18.7^a^	1.4^a^	13.5^b^	27^c^	9^d^	4.5^b^	10.9^c^	1.7^b^	403^b^	273^a^	8.0^d^	2.2^a^	15.9^a^
	Cauliflower	8.2	15.9^b^	1.1^b^	18.3^a^	39^a^	14^a^	5.1^a^	13.7^a^	2.2^a^	439^a^	243^b^	8.4^c^	1.8^b^	14.6^c^
	*Overall mean*	8.2	17.3^A^	1.2^A^	15.9^A^	33^A^	11	4.8^A^	12.3^A^	1.9^A^	421^A^	258^A^	8.2^B^	2.0	15.3^A^
s.e.d.		0.026	0.027	0.04	0.05	0.06	0.02	0.08	0.04	0.02	0.22	0.02	0.01	0.04	0.08
System	1	n.s.	^**^	^**^	^**^	^**^	n.s.	^**^	^**^	^**^	^**^	^**^	^**^	n.s.	^**^
Crop	1	n.s.	^**^	^**^	^**^	^**^	^**^	^*^	^**^	^**^	^**^	^**^	^**^	n.s.	^**^
S × C	1	n.s.	^**^	^**^	^**^	^**^	^**^	^**^	^**^	^**^	^**^	^**^	^**^	^**^	^**^

Different letters stand for statistically significant differences at *p* < 0.05 (Fisher's LSD test).

† %s.s;

†† mg kg^−1^;

††† Cation Exchange Capacity meq 100 g^−1^.

n.s., not significant.

^*^significant at p < 0.05.

^**^significant at p < 0.01.

Regarding mineral availability, under organic management total and nitric nitrogen content increased over time. This is in accordance with Möller (2009) and Watson et al. (2002) who reported that the cycles of carbon (C) and nitrogen (N) are strongly linked, especially in sustainable agroecosystems. It is probably due to the characteristics of the soil N input of the organic system represented by organic fertilizers and green manure. These are more efficient in increasing the soil organic N pool overtime with respect to synthetic fertilizers (Watson et al., 2002; Möller, 2009).

Additionally, K, P, Mg, Mn, Cu, and Zn were found in high concentrations in the organic managed soils; conversely, Fe was not influenced by soil management. Consequently the C/N ratio was higher in conventional system (9.3 vs. 8.2).

Soil microorganisms govern the numerous nutrient cycling reactions in soils; the higher mineral availability in organic systems is a consequence of the higher microorganism activities (Oberson et al., 1993). Phosphorus, potassium, and magnesium fluxes through the microbial biomass is faster in organic soils, and more minerals are normally bound in the microbial biomass (Oberson et al., 1996; Oehl et al., 2001). For all the mineral elements under organic system, except for Mg and N_tot_, areas cropped with cauliflower recorded higher values than fennel and lower values were recorded for organic matter, Mg/K ratio CEC and N_tot_. Under conventional, in cauliflower areas we registered higher values of N- NO_3_, P, Fe, Cu, K, Mg, and Mg/K ratio, while organic matter and C/N ratio were lower.

### Cultivable bacteria and fungi

The effects of long-term impact of soil management and crop production practices on the composition of soil microbial populations were studied. Plate counts are reported as mean values in Table 3. Total mesophilic bacteria were detected with a value of about 5 log CFU/g with the only exception of organic soil cultivated with fennel showing a log higher (6 log CFU/g). Nitrogen fixing bacteria were not affected by the two management systems, while they significantly differed with crop (*p* < 0.05) and management x crop interaction (*p* < 0.05). Several studies have indicated that microbial diversity is affected by crop species and rotation due to differences in root exudation that stimulate microbial growth in the rhizosphere (Wasaki et al., 2005; Micallef et al., 2009). Soil management and crop species significantly affected the number of actinobacteria with the organic treatment showing the highest values. Actinobacteria are involved in organic matter turnover and carbon cycling. They can decompose some recalcitrant carbon sources including cellulose and chitin (Fließbach et al., 2007; Li et al., 2012). Cultivable fungi were <10^2^ CFU/g in both systems even if they are a versatile group able to adapt and grow under extreme environmental conditions (Anand et al., 2006). Probably, culture conditions are very simple and homogeneous, in stark contrast to natural environments. So it is very difficult to quantitatively describe a fungal community using plate counts (Ritz, 2007).

**Table 3:** **Total mesophilic bacteria, nitrogen-fixing bacteria and actinomycetes in soils as affected by long term management (conventional vs. organic) and crop species**.

**System**	**Crop**	***CFU/g***		
		***Total mesophilic bacteria***	***Nitrogen- fixing bacteria***	***Actinomycetes***
Conventional	Fennel	1.4 × 10^5^	1.1 × 10^5^	2.4 × 10^5a^
	Cauliflower	1.2 × 10^5^	1.1 × 10^5^	1.3 × 10^5b^
	*Overall average*	1.3 × 10^5B^	1.1 × 10^5^	1.8 × 10^5B^
Organic	Fennel	1.1 × 10^6^	1.9 × 10^5a^	1.6 × 10^6a^
	Cauliflower	7.0 × 10^5^	7.5 × 10^4b^	1.1 × 10^6b^
	*Overall average*	8.8 × 10^5A^	1.3 × 10^5^	1.3 × 10^6A^
s.e.d.		294289	33750	161618.6
System		^**^	n.s.	^**^
Crop		n.s.	^*^	^*^
S × C		n.s.	^*^	n.s.

Different letters stand for statistically significant differences at *p* < 0.05 (Fisher's LSD test).

n.s., not significant.

^*^significant at p < 0.05.

^**^significant at p < 0.01.

Crop management involves a wide range of practices which can impact on the fungal populations creating conditions either favorable or not to their growth (Menendez et al., 2001; Gosling et al., 2006). In accordance to our results, organic managements may fail to favor fungal communities even after several years (Scullion et al., 1998). Probably, the value of 33 mg/kg P found in organic soil samples was quite high and negatively influenced cultivable fungi growth, as suggested by Dekkers and van der Werff (2001).

### PLFA analysis

The microbial community assessed using a lipid-based approach reflected the cultivable and uncultivable current living community, overcoming the disadvantages associated to conventional systems. Despite fungi not being detected using cultivation methods, fungal and bacterial PLFAs were reported for both microbial groups and are presented in Table 4. PLFAs profiles displayed significant differences between organic and conventional systems. PLFAs biomass was significantly higher in soils subjected to organic management showing values of 13.25 (ORG/FEN) and 10.24 (ORG/CAU) which were at least 8 times higher than those found in conventional soils (2.52 in CON/FEN and 4.37 in CON/CAU) (Table 4). In this study, the interaction between system and crop appeared to have a great impact on soil microbial community structure suggesting that they could generate different and unique ecological niches (Vallejo et al., 2012). Fungal PLFAs (18:1ω9c and 18:2ω6, 9c) were detected almost in all soil samples regardless of crop species. In particular, the fungal biomarker 18:2ϖ6, 9c ranged from 0.53 to 0.59 nmol/g in organic cauliflower and fennel, respectively, while it was absent in conventional soil samples. The second fungal PLFA, 18:1ϖ9c, was present in conventional soils, even if at significant lower concentration than in organic soils (average value 0.54 vs. 1.47, respectively). However, this PFLA is also associated to Gram positive bacteria (Frostegard et al., 1993). Determining the reason for the presence of fungi in organic system is complex because the mechanisms involved in the soil re-colonization and the time required are unknown (Gosling et al., 2006). In this study, long-term organic management positively influenced uncultivable fungal concentration. Probably, the increase in C registered in the organic soil could have led to increased fungal biomass as reported by other authors (Kandeler et al., 1999; Marschner et al., 2003). The concentrations of Gram positive (i15:0, a15:0, i16:0, i17:0, a17:0) and Gram negative (15:0, 17:0, 16:1ω9c, cy17.0, cy19:0) bacterial PLFAs were higher in organic soils, in fact their presence have been related to quality of organic matter. In particular, high proportion of Gram negative bacteria is correlated to a shift from oligotrophic to more copiotrophic conditions in the soil (Yao et al., 2000; Kourtev et al., 2002).

**Table 4:** **Phospholipid fatty acids in soils as affected by long term management (conventional vs. organic) and crop species**.

**System**	**Crop**	***Fatty acids (nmol/g dry weight soil)***													
		**15:0**	**17:0**	**i15:0**	**a15:0**	**i16:0**	**i17:0**	**a17:0**	**16:1w9c**	**Cy17:0**	**18:1w9c**	**Cy19:0**	**18:2w6, 9c**	**Total**	**F/B**
Conventional	Fennel	0	0.20^c^	0.32^d^	0.15^d^	0.13	0.22	0.20	0.42	0.17^d^	0.49	0.22	0	2.52^d^	0.24
	Cauliflower	0	0.24^c^	0.83^c^	0.47^c^	0.26	0.31	0.35	0.66	0.40^c^	0.58	0.27	0	4.37^c^	0.15
	*Overall average*	0^B^	0.22	0.58^B^	0.31^B^	0.20^B^	0.27^B^	0.28^B^	0.54^B^	0.29^B^	0.54^B^	0.25^B^	0^B^	3.48^B^	0.18
Organic	Fennel	0.32	0.40^a^	3.02^a^	1.68^a^	0.84	0.72	0.71	2.21	0.73^a^	1.48	0.55	0.59	13.25^a^	0.18
	Cauliflower	0.21	0.28^b^	1.81^b^	1.03^b^	0.68	0.62	0.69	1.60	0.67^b^	1.45	0.67	0.53	10.24^b^	0.23
	*Overall average*	0.27^A^	0.34	2.42^A^	1.36^A^	0.76^A^	0.67^A^	0.70^A^	1.91^A^	0.70^A^	1.47^A^	0.61^A^	0.56^A^	11.77^A^	0.21
System		^*^	n.s.	^*^	^*^	^**^	^*^	^*^	^*^	^*^	^**^	^**^	^**^	^**^	
Crop		n.s.	n.s.	n.s.	n.s.	n.s.	n.s.	n.s.	n.s.	n.s	n.s.	n.s.	n.s.	n.s	
S x C		n.s.	^*^	^*^	^*^	n.s.	n.s.	n.s.	n.s.	^*^	n.s.	n.s.	n.s.	^*^	

Different letters stand for statistically significant differences at *p* < 0.05 (Fisher's LSD test).

^*^significant at p < 0.05.

^**^significant at p < 0.01.

n.s., not significant.

F/B, Fungal/bacterial ratio.

Total anaerobic bacteria (cy17:0 and cy19:0 biomarkers) were influenced by soil management and were significantly higher in samples collected from soil fertilized with green manure and organic fertilizer. Moreover, cy17:0 specific biomarker of sulfate reducing bacteria (*Desolphobacter* sp.), within the conventional system, was influenced by the crop with values of 0.17 in CON/FEN and 0.40 in CON/CAU (Table 4). In the conventional system the highest percentages of microbial biomarkers were found in soils cultivated with cauliflower, while in organic system in those cultivated with fennel, suggesting that the interaction between system and crop exerts a strong effect in shaping soil microbial community structure.

Comparison of Fungal/Bacterial ratio (F/B) is an indicator of changes in the relative abundance of these two microbial groups (Vallejo et al., 2012). In the conventional system the obtained values ranged from 0.15 (CON/CAU) to 0.24 (CON/FEN), while in the organic one they were 0.23 (ORG/CAU) and 0.18 (ORG/FEN) (Table 4). Generally, high values are reported in the soils subjected to organic management, and they are inversely proportional to N application rates (Kong et al., 2011). The relatively low F/B ratios suggested the dominance of bacterial PLFAs in all soil samples which may have resulted from the low C/N ratio in both management systems as reported in Table 2.

### Determination of fungi by PCR-DGGE

To develop a deeper understanding of “soil fungi sociology” in organic and conventional soils PCR-DGGE analysis was performed. It is well known that PCR-DGGE analysis represents a powerful tool to profile the structure of microbial communities in environmental samples without cultivation and to highlight dominant microbial populations in response to environmental variations.

The obtained DGGE fingerprinting showed few definite bands for all the soil samples suggesting the presence of a limited number of dominant, ubiquitous and ecologically well-adapted fungi types (data not shown).

**Figure 1:**
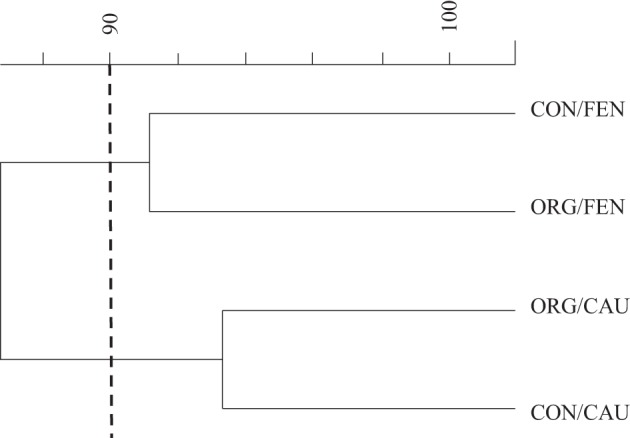
**Cluster analysis of banding patterns generated by PCR-DGGE**.

DGGE dendrogram obtained by analyzing the amplified 18S rDNA fragments is shown in Figure 1. Using an arbitrary similarity level of 90% two clusters can be identified and the fingerprints clustered according to the crop instead of management system.

Selected DGGE bands were excised and re-amplified. The sequenced bands corresponded to two uncultivable fungi and a further eight fungal species as reported in Table 5. Moreover, a great number of faint bands indicated that many equally abundant populations characterized all soil samples. The sequence of these bands displayed similarity values lower than 85% to reference sequences, so they were not considered in the analysis.

**Table 5:** **Identification of bands in DGGE profiles of fungal population from soils affected by long term management (conventional vs. organic) and crop species**.

**Closest relative**	**% Identity**	**Accession number**	**Isolation source**
*Arthrobotrys oligospora*	99	AJ001987	ORG/FEN, ORG/CAU,
*Paecilomyces lilacinus*	100	GU980027	CON/FEN, CON/CAU
*Glomus versiforme*	99	EU164972	
Uncultured fungus	98	FM202457	
*Gigaspora gigantea*	98	EF014362	
*Sebacina* spp.	98	HQ215802	CON/CAU
*Glomus mossae*	100	NG017178	ORG/FEN, ORG/CAU,
Uncultured soil fungus	98	FM202450	CON/FEN, CON/CAU
*Glomus* sp.	98	FR847093	
*Acremonium kiliense*	100	U43973	ORG/FEN, ORG/CAU, CON/FEN

The arbuscular mycorrhizal fungus of the *Glomus* genus dominated in all soil samples. Its dominance has been reported in several ecosystems, ranging from forest (Wubet et al., 2003) to highly disturbed agricultural fields (Daniell et al., 2001). Only a member of *Diversisporales* (*Gigaspora gigantea*), which generally decreases with cultivation (Gosling et al., 2006), was detected.

Obtained results suggested a relationship between soil resilience and biodiversity as reported also by other authors (Elliott and Lynch, 1994). The absence of a high fungal diversity in organic soils is probably due the fact that long-term or chronic stresses could eliminate part of the microbial community selecting a small number of species tolerant of intensive farming practices. There are no available data indicating the mechanisms and the time required for re-colonization of agricultural land and our study indicated that 10 years of organic management are not enough to increase fungal biodiversity.

### PCA analysis

**Figure 2:**
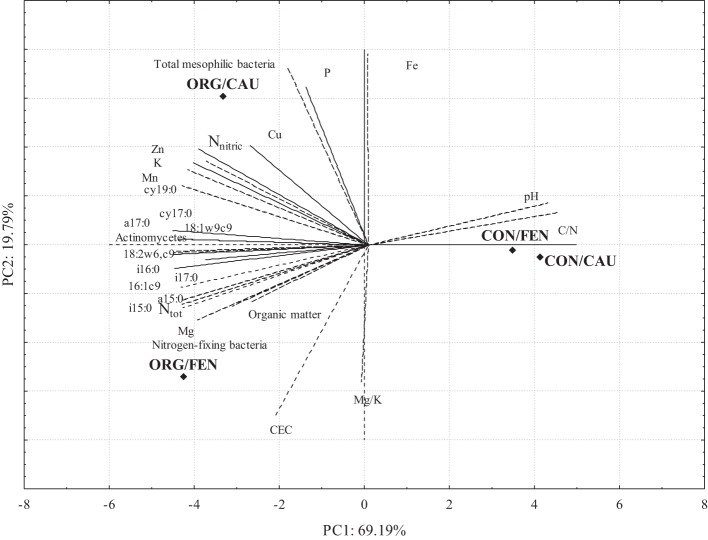
**Two principal component analysis (PCA) correlation bi-plot: rhombus stand for the standardized scores on PC1 and PC2 of four soil management/crop combinations (ORG/CAU, ORG/FEN, CON/CAU, CON/FEN): lines represent the correlation between standardized variables (PLFAs, chemical and microbiological characteristics of soils) and PCs**.

The results of PCA analysis are shown in Figure 2; PC1 and PC2 captured 88.98% of the total data variability. The effect of management system clearly emerges from the bi-plot analysis: indeed, CON is distinctively separated by PC1 with respect to ORG, independently to the crop. While in CON the values of PCs are similar between fennel and cauliflower, in ORG system the two crops are separated by PC2. Cu, N nitric, Zn, K, Mn, P, aerobic mesophilic bacteria and cy19:0 constitute a group well associated to ORG/CAU while organic matter, CEC, Mg, N_tot_, nitrogen fixing bacteria, i15:0, a15:0, i17:0, 16:1w9c, i16:0, and 18:2w6, 9c seems more associated with ORG/FEN.

Conventional soil samples are strictly correlated independently from the crop, in fact they are characterized by similar values of C/N, N_tot_, organic matter, Mn and Zn. On the contrary the other two sites ORG/FEN and ORG/CAU are quite different. ORG/FEN is characterized by an higher content of organic matter, CEC, N_tot_ and Mg. ORG/CAU is well differentiated from the others because of the content in P, K, Fe, Cu and Zn. This statistical approach confirmed that the crop influenced more the organic system that the conventional one.

## Conclusions

This study contributes to the understanding of soil fertility status and microbial relationships with long-term soil managements (organic and conventional). Organic management showed higher value of organic matter, N as well as some important macro and micronutrients for crop growth. No cultivable fungi were detected in both systems, but PLFA analysis revealed an higher fungal biomass in organic management confirming the fundamental importance of C for soil microbial biomass development. Moreover, PCR-DGGE analysis revealed a low fungal biodiversity suggesting that long-term or chronic stresses could eliminate part of the microbial community which is difficult to be restored.

Further studies based on –omic approaches should be addressed to better understand the effects of different soil managements on soil fungal and bacterial functionality and community structure providing useful information for the development of more sustainable agricultural practices.

### Conflict of interest statement

The authors declare that the research was conducted in the absence of any commercial or financial relationships that could be construed as a potential conflict of interest.
